# ML-792 impairs *Trypanosoma brucei* growth through SUMO pathway disruption: toward E1 as a candidate antitrypanosomal target

**DOI:** 10.3389/fcimb.2026.1850640

**Published:** 2026-06-19

**Authors:** Emily Denise Fischman, Melina Serassio, Vanina Eder Alvarez, Paula Ana Iribarren

**Affiliations:** 1 Instituto de Investigaciones Biotecnológicas, Universidad Nacional de San Martín (UNSAM) - Consejo Nacional de Investigaciones Científicas y Técnicas (CONICET), San Martin, Argentina; 2 Escuela de Bio y Nanotecnologías (EByN), Universidad Nacional de San Martín, San Martin, Argentina

**Keywords:** inhibition, ML-792, SAE, SUMOylation, *Trypanosoma brucei*

## Abstract

African trypanosomiasis is a neglected disease affecting humans and animals in sub-Saharan Africa, caused by extracellular parasites of the *Trypanosoma brucei* species complex. Despite recent progress in disease control, current treatments are limited by toxicity, emerging resistance, and incomplete understanding of their mechanisms of action, highlighting the need for new targeted therapeutic strategies. In *Trypanosoma brucei*, post-translational modifications such as SUMOylation play essential roles in cellular processes, including cell cycle progression and variable surface antigen expression, making this pathway an attractive therapeutic target. We evaluated the effect of ML-792, a selective inhibitor of the SUMO-activating enzyme (E1, SAE), on bloodstream form parasites. Structural analysis showed conservation of key features of the E1 enzyme, consistent with susceptibility to inhibition. ML-792 treatment reduced global SUMO conjugation and nuclear SUMO levels and impaired parasite proliferation in a dose-dependent manner, with an IC_50_ in the micromolar range, and induced defects in cell cycle progression, including abnormal nuclei/kinetoplast configurations. These results demonstrate that pharmacological inhibition of SUMOylation disrupts essential processes in *T. brucei* and support the SUMO pathway as a potential target for therapeutic intervention.

## 1 Introduction

African trypanosomiasis is a neglected disease affecting both humans and animals in sub-Saharan Africa, caused by extracellular protozoan parasites of the *Trypanosoma brucei* species complex. *T. brucei gambiense* and *T. brucei rhodesiense* are responsible for human African trypanosomiasis (HAT), or sleeping sickness, whereas *T. brucei brucei*, together with *T. congolense* and *T. vivax*, causes animal African trypanosomiasis (AAT), also known as Nagana (Steverding, 2017). Transmission occurs primarily through the bite of infected tsetse flies (*Glossina* spp.), and the parasite alternates between insect and mammalian hosts, adopting distinct developmental stages adapted to each environment. In the mammalian host, bloodstream form (BSF) parasites proliferate extracellularly in blood and tissues, where they are continuously exposed to the host immune system. In humans, clinical manifestations vary depending on the infecting strain, ranging from acute to chronic conditions that may progress to severe neurological involvement and death (Buscher et al., 2017). In animals, infection is associated with weight loss, reproductive disorders, and decreased productivity (Pereira et al., 2024). Despite the substantial reduction in reported HAT cases in recent years, the disease remains a public health concern, while AAT continues to impose a major economic burden, affecting millions of livestock and causing significant losses in agricultural productivity (Abro et al., 2021; Habeeb et al., 2021; Tora and Dana, 2024). Current treatments are limited by toxicity, emerging resistance, and incomplete knowledge of their mechanisms of action, highlighting the need for new, targeted therapeutic strategies (Venturelli et al., 2022).

In *T. brucei*, gene expression is primarily regulated at the post-transcriptional level, placing particular importance on post-translational modifications (PTMs) as key modulators of protein function. Among these, SUMOylation has emerged as a central regulatory mechanism in parasite biology. This reversible modification involves the covalent attachment of the small ubiquitin-like modifier (SUMO) to target proteins and has been shown to be essential in the parasite (Liao et al., 2010; Obado et al., 2011). Proteomic studies have identified numerous SUMO substrates involved in fundamental cellular processes, including DNA replication and repair, chromatin organization, transcriptional regulation, and cell cycle progression (Iribarren et al., 2015a). In BSF parasites, protein SUMOylation plays a key role in antigenic variation and host immune evasion by supporting the monoallelic and switchable expression of variant surface antigens. This process is associated with a specialized nuclear structure, the highly SUMOylated focus (HSF), where SUMOylation mediates the recruitment of RNA polymerase I (Pol I) and promotes transcription at the active VSG expression site (Lopez-Farfan et al., 2014).

Given its central role in essential cellular processes and parasite-specific regulatory pathways, the SUMOylation machinery represents an attractive target for therapeutic intervention. SUMO conjugation is mediated by a conserved enzymatic cascade involving an activating enzyme (E1), a single conjugating enzyme (E2, Ubc9), and, in some cases, E3 ligases that enhance substrate specificity. This process is reversible and tightly regulated by SUMO-specific proteases, which are responsible for both SUMO maturation and deconjugation from target proteins (Flotho and Melchior, 2013).

**Figure 1:**
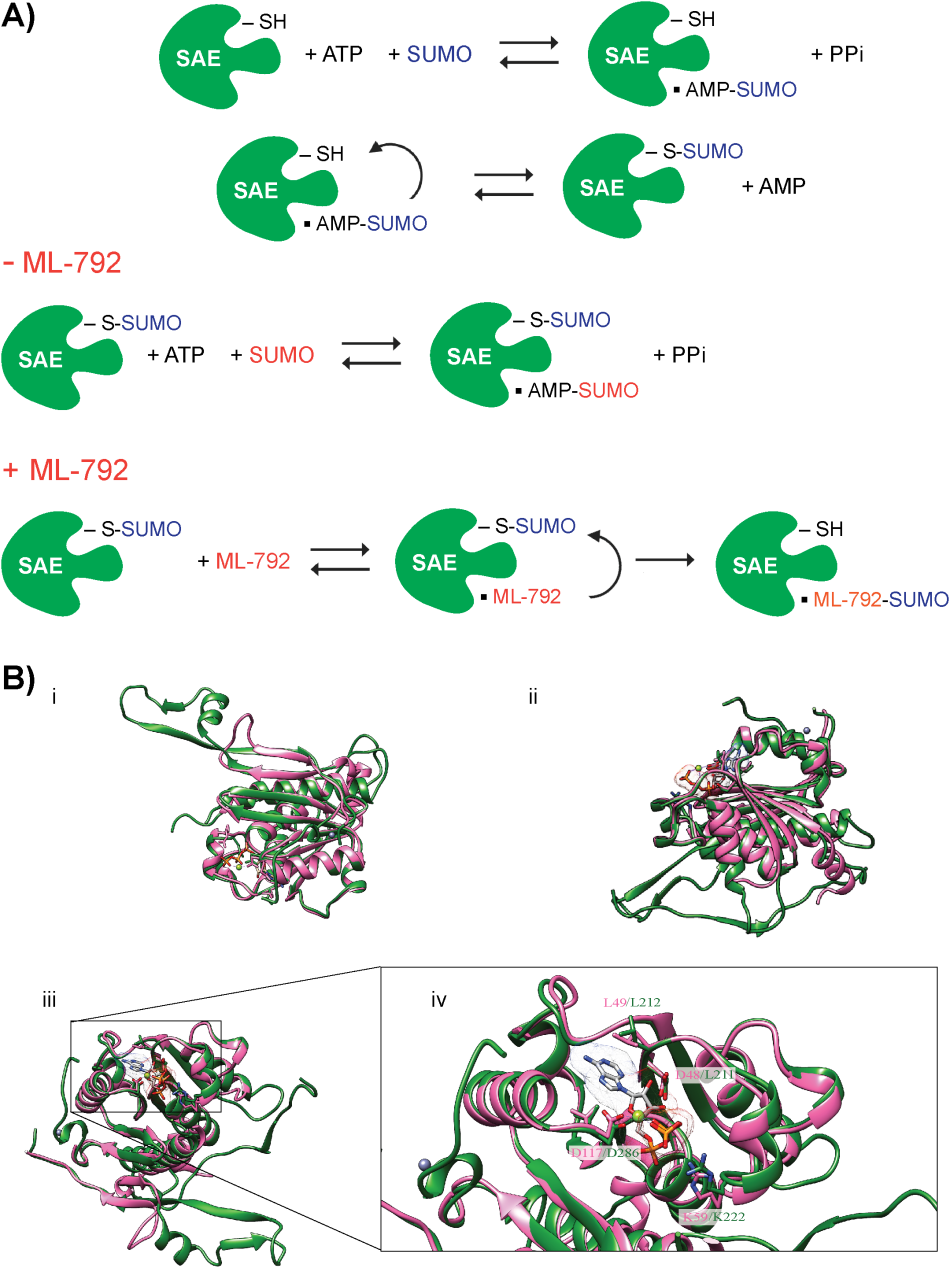
**(A)** Schematic representation of the reaction mechanism for SUMO activation and formation of the ML-792–SUMO adduct. Initially, Mg·ATP and SUMO bind to the enzyme to yield SUMO-AMP, releasing inorganic pyrophosphate (PPi). SUMO-AMP then reacts in the SAE2 active site with the thiol group of the catalytic cysteine, forming a thioester bond and releasing AMP. In the absence of inhibition (−ML-792), a second ATP and SUMO bind to the enzyme to form a second SUMO-AMP in the adenylation domain, resulting in a ternary complex with two SUMO molecules bound to the enzyme. This form of SAE2 is capable of transthiolation of SUMO to the E2 enzyme. In contrast, in the presence of the inhibitor (+ML-792), this process is blocked as ML-792 binds to the nucleotide binding pocket of the SAE2–SUMO thioester form and reacts with thioester-bound SUMO to form the ML-792-SUMO adduct. Upon formation, the adduct occupies the SAE2 adenylation domain, preventing SUMO conjugation. **(B)** (i-iii) Ribbon diagrams showing the structural superposition of the adenylation domains from *T. brucei* (green) and human (pink) SAE2 subunits in complex with Mg·ATP. The *T. brucei* model was generated using AlphaFold3, while the human ortholog structure was obtained from the PDB (ID: 1Y8Q). (iv) Magnified view of the adenylation/ATP-binding pocket, highlighting the high structural conservation of the residues coordinating the Mg·ATP complex. Molecular graphics and structural superpositions were performed using UCSF Chimera (Global RMSD: 1.571).

Among the inhibitors developed against this pathway, ML-792 has emerged as a potent and selective inhibitor of the SUMO-activating enzyme (E1, SAE). Mechanistically, ML-792 acts as an adenosine sulfamate analog that forms a covalent adduct with SUMO in the active site of the E1 enzyme, mimicking the SUMO–adenylate intermediate and thereby blocking further catalytic turnover (**Figure 1A**). This results in effective inhibition of SUMO transfer to downstream substrates. ML-792 displays high specificity for SUMO E1 over other ubiquitin-like activating enzymes and has shown strong antiproliferative activity in tumor cell lines, with limited effects on non-dividing cells (He et al., 2017; Garcia et al., 2021). However, whether this inhibitory mechanism is conserved in trypanosomatids, and to what extent SUMO pathway inhibition can be exploited therapeutically in these organisms, remains to be established.

Given the conservation of the SUMOylation machinery between humans and *T. brucei*, and the central role of this pathway in parasite biology, we hypothesized that pharmacological inhibition of SUMO activation could impair parasite viability. In this study, we evaluated the effect of ML-792 on bloodstream form parasites, analyzing its impact on SUMO conjugation, subcellular SUMO distribution, and parasite proliferation. Our findings highlight the SUMO pathway as a possible target in *T. brucei* and support its potential for the development of new therapeutic strategies against African trypanosomiasis.

## Materials and methods

### Protein structure modeling and comparison

The structure of SAE2/UBA2 (Tb927.5.3430) from *T. brucei* was modeled using AlphaFold3 (seed: 1) (Abramson et al., 2024), using the protein sequence deposited in TriTrypDB.

For structural comparison, the E1 enzyme structure (PDB ID: 1Y8Q) from *H. sapiens*, available in the Protein Data Bank (PDB) (Lois and Lima, 2005), and the top-ranked SAE2/UBA2 model from *T. brucei* generated by AlphaFold3 were used. The structures of both SAE2 domains were compared by performing a structural alignment using UCSF Chimera 1.19 with default parameters.

### Reagents

The inhibitor ML-792 (98% purity) was acquired from A2B Chem LLC (San Diego, United States). The solid drug was resuspended in DMSO (Merck, Darmstadt, Germany) at a stock concentration of 10 mM.

### Parasite culture

*Trypanosoma brucei* bloodstream form “Single Marker” (SM) parasites (T7RNAP TETR NEO) (Alibu et al., 2005) were grown at 37 °C and 5% CO_2_ in HMI-9 medium (Hirumi and Hirumi, 1989) (Life Technologies, Carlsbad, CA, USA), supplemented with 10% (vol/vol) heat-inactivated fetal calf serum (Natocor, Córdoba, Argentina) and 2.5 µg/ml G-418 (InvivoGen, San Diego, CA, USA). Doubling time was calculated by counting cell numbers daily in quadruplicate using a Neubauer hemocytometer. For RNA polymerase I visualization, SM parasites were transfected with pENT5-Halo::RPA2 as previously described (Budzak et al., 2022).

### Determination of IC_50_ from ML-792 *in vitro*

To determine the IC_50_ of ML-792, 10^4^ parasites were seeded in a 96-well plate. Cells were treated with serial dilutions of the drug in a final volume of 100 µl and incubated at 37 °C for 72 hours. Inhibitor concentration ranged from 0.01 µM to 10 µM. A 10mM stock solution was prepared in 100% DMSO and diluted in culture medium to achieve the desired drug concentrations with a uniform final DMSO concentration of 0.5% (v/v) in all wells, a concentration previously established as non-toxic to the parasite under these experimental conditions. After 68 hours of incubation, samples were transferred to a black 96-well plate (Greiner Bio-One GmbH, Frickenhausen, Alemania), and the resazurin assay was performed. Briefly, 11 µl of a 4 mM stock solution of resazurin was added per well (final resazurin concentration 44 µM). After incubating the parasites at 37 °C for 4 hours, fluorescence resulting from resazurin reduction by viable parasites was measured using the FilterMax F5 plate fluorometer (λ_ex = 535 nm; λ_em = 595 nm) (Sykes and Avery, 2009). All concentrations were tested in triplicate, including controls. Control wells included medium without parasites (0% viability) and wells where parasites were treated with 0.5% DMSO, the drug vehicle (100% viability). Fluorescence measurements were normalized to the average of the triplicates treated with 0.5% DMSO, allowing calculation of viability percentages for each inhibitor concentration. These percentages were then plotted as a function of the base-10 logarithm of the corresponding concentration. The concentration resulting in 50% viability corresponds to the IC_50_, which was calculated using GraphPad Prism 9 by fitting the data to a dose-response curve with the variable slope model (four parameters). The IC_50_ and its error were calculated by taking the base-10 logarithm of the IC_50_ for independent experiments.

### Generation of anti-*Tb*SUMO polyclonal antibodies

Male BALB/c mice were immunized intraperitoneally with 20 µg of purified recombinant 6xHis-*Tb*SUMO. Following the initial immunization, two booster doses of 10 µg each were administered at 2 and 4 weeks. One week after the second booster, an exploratory blood collection was performed to confirm antibody titer. Finally, mice were euthanized, and the collected antisera were utilized for Western blot and indirect immunofluorescence assays.

### Electrophoresis and immunoblotting

Parasites at a density of 1.5 × 10^5^ cells/ml were incubated with 5 µM ML-792 or 0.5% DMSO for 48 hours. Cells (3 × 10^7^) were then harvested by centrifugation (1,000 × g for 10 min), resuspended in Laemmli sample buffer (0.125 M Tris, pH 6.8, 4% SDS [w/v], 20% glycerol [v/v], 0.02% bromophenol blue [w/v], 100 mM DTT), and boiled for 5 minutes. Total protein extracts were resolved by SDS-PAGE according to the technique described by Laemmli (1970) and subsequently transferred to a nitrocellulose Hybond ECL membrane (GE Healthcare, Pittsburgh, PA, USA) using a semi-dry method at 0.8 mA/cm^2^ for 90 minutes. The membrane was incubated for 1 hour in 1% non-fat milk in TBS (50 mM Tris-HCl, pH 7.6, 150 mM NaCl), then probed overnight at 4 °C with polyclonal mouse anti-*Tb*SUMO antibody (1:500) or rabbit anti-PABP-C antibody (1:1000) (D'Orso and Frasch, 2002). Alexa Fluor 680 AffiniPure goat anti-mouse IgG (H+L) or Alexa Fluor 790 AffiniPure goat anti-rabbit IgG (H+L) secondary antibodies (Jackson ImmunoResearch Laboratories, West Grove, PA, USA), diluted 1:20,000, were detected using an Odyssey laser-scanning system (LI-COR Biosciences, Lincoln, NE, USA).

### Indirect immunofluorescence and cell cycle analysis

Parasite cultures at 1.5 × 10^5^ cells/ml were treated as previously described with 5 µM ML-792 or 0.5% DMSO for 24 or 48 hours. Approximately 3–5 million cells were harvested by centrifugation (1000 × g for 10 min), fixed with 2% paraformaldehyde in phosphate-buffered saline (PBS) for 20 minutes, and washed twice with PBS. Parasites were attached to glass coverslips previously treated with poly-L-lysine (Merck) for 30 minutes and then incubated with 25 mM NH_4_Cl for 15 min. Cells were permeabilized with 0.1% Triton X-100 in PBS for exactly 10 minutes. After three PBS washes, samples were incubated with blocking solution (3% BSA, 5% normal goat serum in PBS) for 1 hour. Polyclonal mouse anti-*Tb*SUMO antibody (1:250 in 1% BSA: PBS) was used as primary antibody. After washing with PBS, coverslips were incubated for 1 hour with polyclonal goat anti-mouse Alexa Fluor 488 (Jackson) diluted 1:1000 in 1% BSA: PBS and 4,6-diamidino-2-phenylindole (DAPI) (Life Technologies). Finally, coverslips were extensively washed and mounted using FluorSave reagent (Merck). Parasites with endogenously tagged Halo::RPA2 were labeled with 200 nM Janelia Fluor 552 HaloTag ligand (JF552; Promega, Madison, WI, USA) in HMI-9 medium for 30 min at 37 °C prior to fixation. A C- Plan-Apochromat 63x/1.4 Oil DIC M27 objective was employed for Airyscan imaging attached to a LSM 980 system. Fluorophores were excited using diode laser lines at 405, 488 and 561 nm, selected according to probe spectral properties. Acquisition settings, including laser power, dwell time and detector gain, were optimized to maximize dynamic range while avoiding pixel saturation. For increased spatial resolution, the LSM 980 was operated with the Airyscan 2 detector in Super-resolution (SR) mode. Spatial sampling in x, y and z followed the Nyquist criterion, with a pixel size of 75.9 nm and a z-step of 0.1 μm. Airyscan image processing was performed in ZEN Blue (v 3.6). When required, images were further deconvolved using the Airyscan Joint Deconvolution method with default parameters. Image analysis was performed using ImageJ.

For cell cycle analysis, parasites were counted and classified based on the number and morphology of their nucleus (N) and kinetoplast (K): G1 (1N1K), S (1N1Ke, elongated kinetoplast), G2/M (1N2K), and post-M/cytokinesis (2N2K) (Wheeler et al., 2013). Parasites with a reconfigured N/K pattern that did not match any of these categories were considered abnormal. Samples were analyzed with an Eclipse 80i microscope (Nikon, Shinagawa, Japan).

To analyze nuclear SUMO signal intensity, regions of interest (ROIs) were defined to outline only the contour of each nucleus by setting a signal threshold in the DAPI channel. ROIs corresponding to the kinetoplast or other high-signal points were excluded. After obtaining the final ROIs, the integrated density values for the SUMO signal were recorded. These results were analyzed in GraphPad Prism 9 to generate violin plots, and the difference between the means for ML-792 and DMSO treatments was evaluated using the Student t-test.

## Results

### Structural conservation of the SUMO E1 enzyme supports susceptibility to ML-792

The SUMO-activating enzyme (E1, SAE) is a heterodimer composed of SAE1 and SAE2 (UBA2), where UBA2 is the catalytically active subunit responsible for SUMO adenylation, thioester bond formation, and transfer to the E2 enzyme Ubc9, while SAE1 contributes to SUMO recognition and structural stability (Lois and Lima, 2005; Schulman and Harper, 2009). To assess whether *T. brucei* SAE2 could be targeted by ML-792, we compared its structure to the human ortholog using an AlphaFold3-predicted model. The overall fold, including the adenylation domain and catalytic cysteine, was well conserved, and key features of the active site were preserved (**Figure 1B**), supporting the potential binding of ML-792 to the parasite enzyme.

### ML-792 exhibits trypanocidal activity against *T. brucei* bloodstream forms

We next determined the trypanocidal potential of ML-792 by measuring its potency against BSF parasites using a resazurin-based viability assay. Parasites were incubated for 72 hours with increasing concentrations of the compound spanning the low nanomolar to micromolar range, and fluorescence values were normalized to those obtained from 0.5% DMSO-treated controls, which were set as 100% viability.

**Figure 2:**
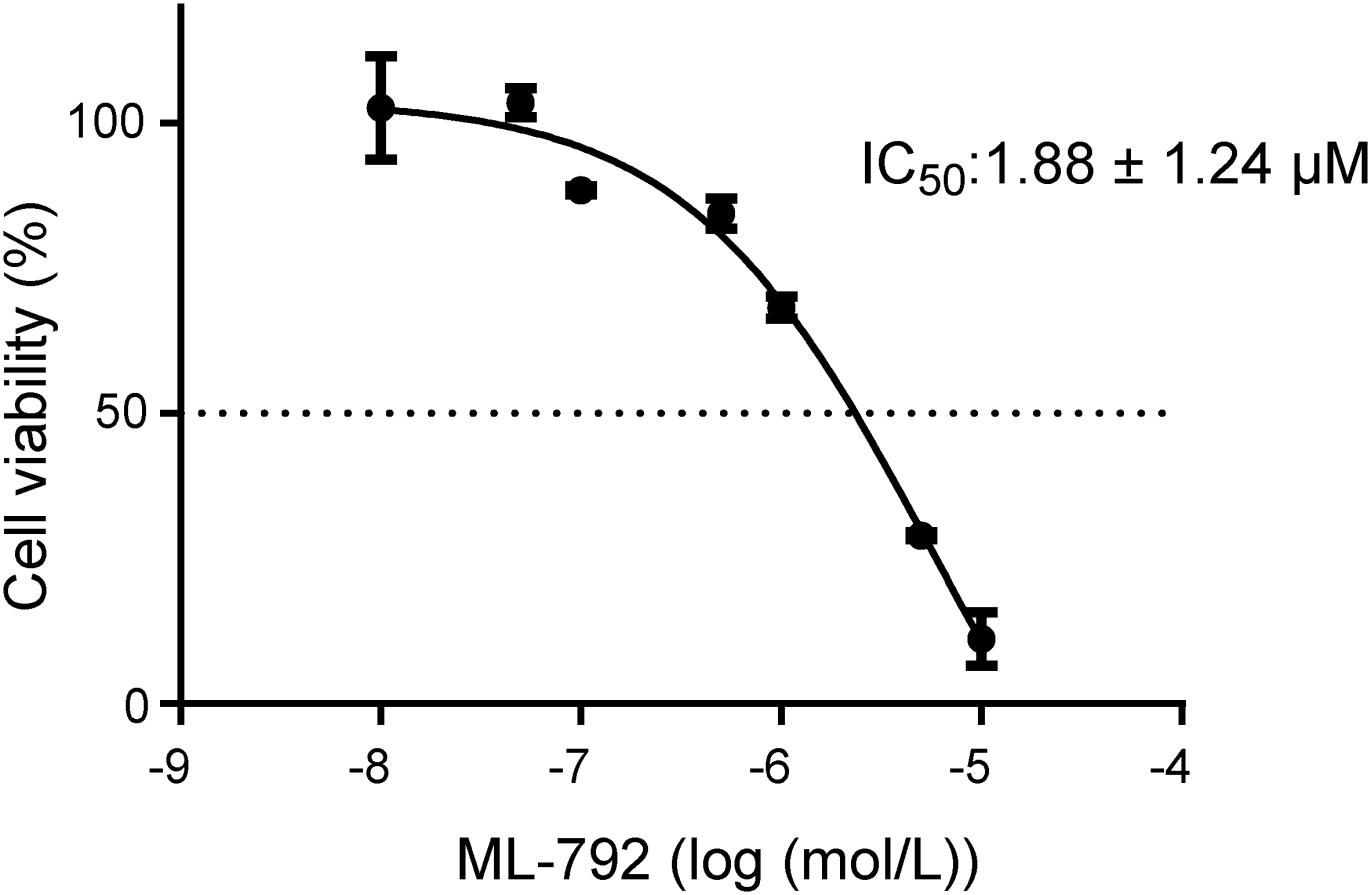
Dose–response curve of ML-792 on BSF parasites. Viability was determined by a resazurin assay and fluorescence measurement at 595 nm. Percentages were normalized to 0.5% DMSO. Results were obtained from three independent biological replicates. The IC_50_ was calculated with GraphPad Prism 9 and fitted to a dose–response curve using the variable slope model (four parameters).

ML-792 reduced parasite proliferation in a dose-dependent manner, producing a sigmoidal response curve characteristic of pharmacological inhibition (**Figure 2**). Nonlinear regression analysis yielded an IC_50_ of 1.88± 1.24 μM. Notably, this value is within the same order of magnitude as that reported for the veterinary trypanocidal drug diminazene aceturate (Dofuor et al., 2019), supporting the activity of ML-792 against *T. brucei* BSF under *in vitro* conditions.

### ML-792 inhibits global SUMO conjugation in *T. brucei*

Based on the IC_50_ value, a working concentration of 5 μM (~2–3× IC_50_) was selected to ensure robust pathway inhibition while maintaining parasite viability for downstream analyses. To determine whether ML-792 interferes with the SUMO conjugation pathway in *T. brucei*, BSF parasites were incubated for 48 hours with the inhibitor or DMSO as a control, and whole-cell extracts were analyzed by Western blot using anti-*Tb*SUMO antibodies (**Supplementary Figure 1**).

**Figure 3:**
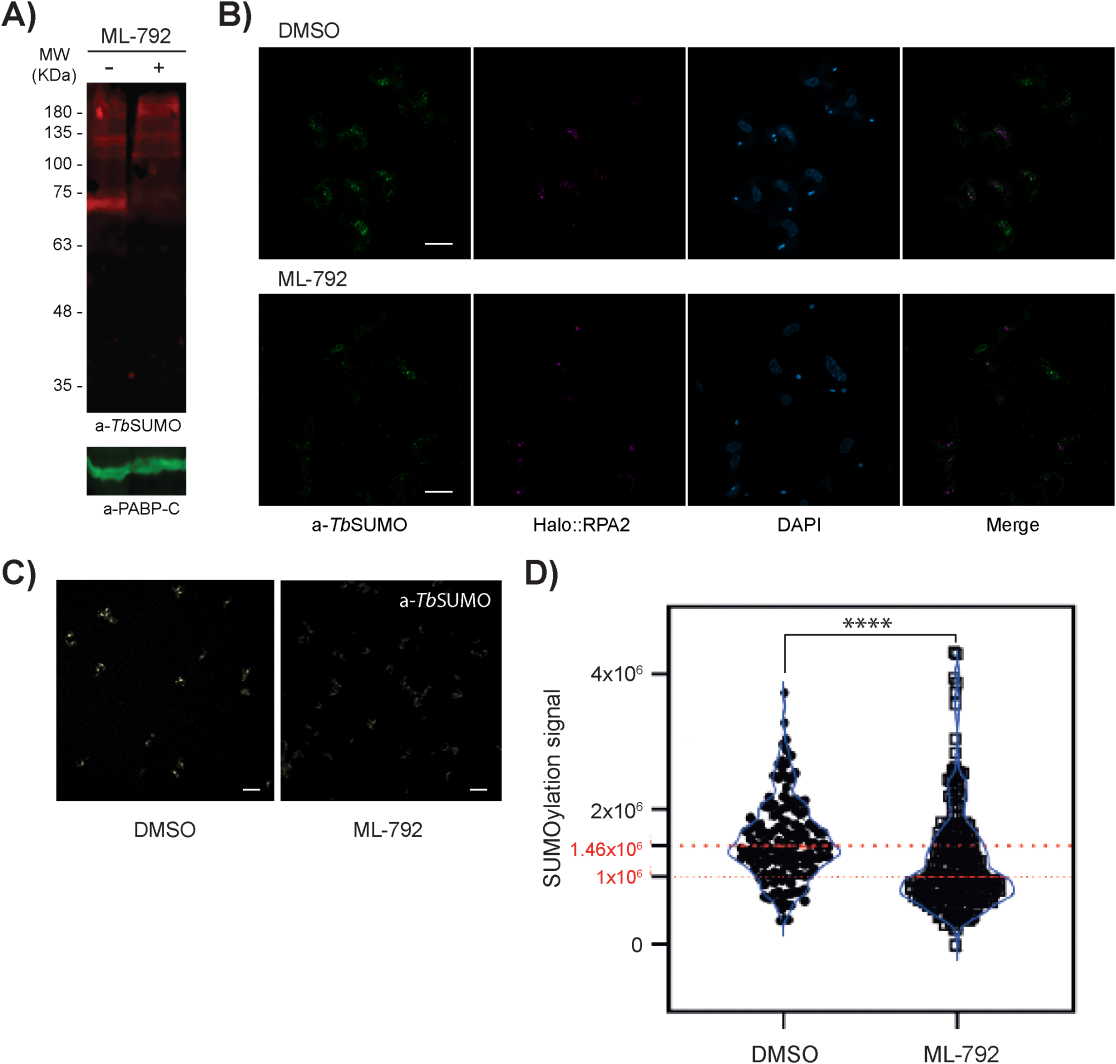
Inhibition of SUMOylation by ML-792. **(A)** BSF parasites were incubated for 48 h in the presence (+) or absence (−) of 5 µM ML-792. Whole-cell extracts were boiled in Laemmli sample buffer immediately after harvesting. Proteins were separated by 7.5% SDS-PAGE (1x10^7^ cells/lane), and SUMO conjugates were analyzed by Western blot using anti-*Tb*SUMO antibodies. Anti-PABP-C antibodies were used as loading control. **(B)** Immunofluorescence analysis of SM-Halo::RPA2 parasites after incubation with 5 µM ML-792 or DMSO for 48 h. Nuclear and kinetoplast DNA were visualized by DAPI staining (blue). Representative images of anti-*Tb*SUMO (green), Halo::RPA2 (magenta), and merged images are shown. The HSF (arrow), the ESB (arrowhead) and the nucleolar Halo::RPA2 (asterisk) are indicated. Scale bars 5 µm. **(C)** Immunofluorescence analysis of SM parasites using anti-*Tb*SUMO antibodies following treatment with 5 µM ML-792 or DMSO for 24 h. Scale bars 10 µm. **(D)** Violin plot comparing the average SUMO signal shown in **(C)** in the parasites’ nuclei (red lines) after treatment with ML-792 (n = 190) or DMSO (n = 130). Statistical analysis: Student’s t-test, ****P < 0.0001.

Control samples displayed a characteristic pattern of high molecular weight SUMO conjugates, reflecting the presence of SUMO-modified substrates. In contrast, ML-792-treated parasites showed a marked reduction in the intensity of these species (**Figure 3A**; **Supplementary Figure 2**), consistent with impaired SUMO conjugation.

### ML-792 reduces nuclear SUMO levels while preserving the highly SUMOylated focus

To further characterize the cellular consequences of SUMO pathway inhibition, we analyzed SUMO distribution by indirect immunofluorescence. In DMSO-treated parasites, SUMO localized predominantly to the nucleus, showing a diffuse nucleoplasmic pattern and a prominent highly SUMOylated focus, as previously reported (Lopez-Farfan et al., 2014). RNA polymerase I, visualized via a HaloTag fusion, displayed both nucleolar localization and a distinct extranucleolar focus corresponding to the expression site body (ESB) (Navarro and Gull, 2001), the site of monoallelic VSG expression, which colocalized with the HSF (Lopez-Farfan et al., 2014).

Immunofluorescence analysis showed that ML-792 treatment led to a noticeable reduction in overall nuclear SUMO signal intensity, consistent with the decrease in SUMO conjugates detected by Western blot. Despite this global reduction, the HSF and the ESB remained detectable in the majority of 1N1K cells (**Figures 3B, C**).

To quantitatively validate these observations, regions of interest were defined based on DAPI staining and nuclear SUMO signal intensity was measured. This analysis confirmed a significant decrease in nuclear SUMO levels in ML-792-treated parasites compared to controls (**Figure 3D**).

### ML-792 impairs cell cycle progression in *T. brucei*

**Figure 4:**
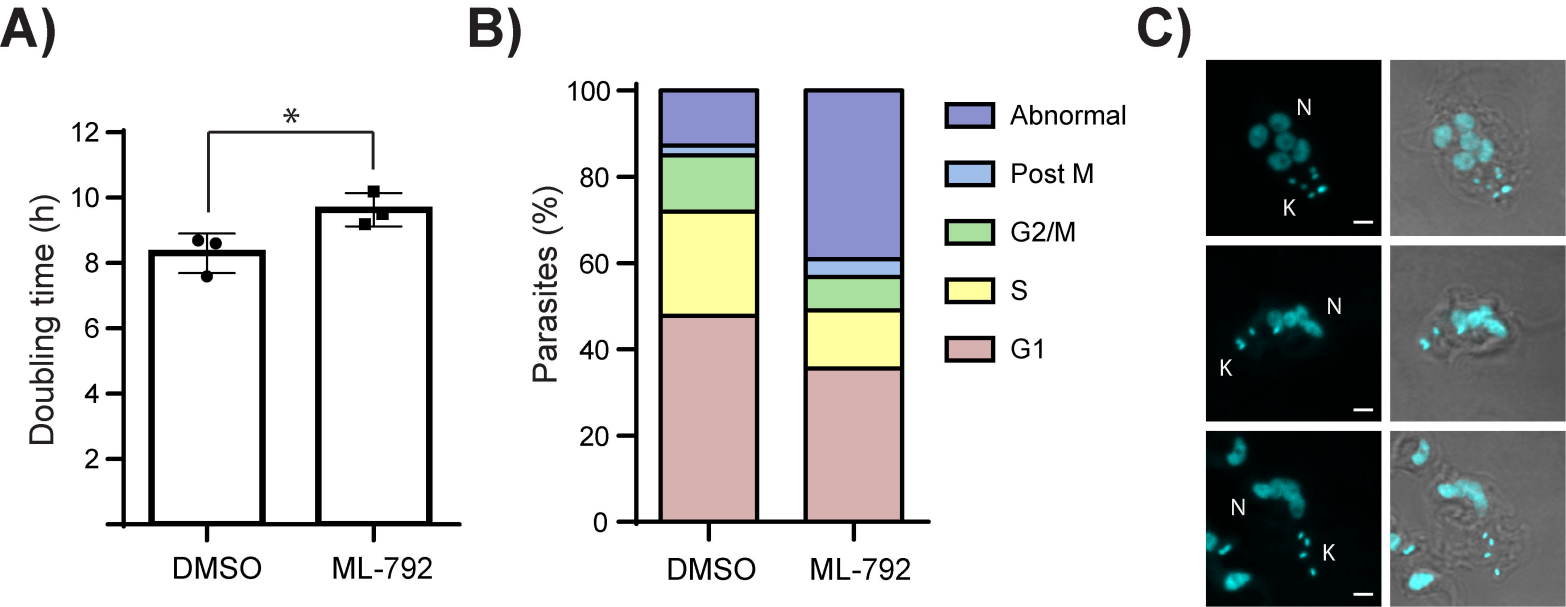
Parasite proliferation and cell cycle analysis after ML-792 treatment. **(A)** SM parasites were cultivated in the presence of 5 µM ML-792 or DMSO to determine doubling time. **(B)** SM parasites incubated with 5 µM ML-792 or DMSO for 24 h were fixed and stained with DAPI for analysis of nucleus and kinetoplast configuration. At least 300 cells were scored. **(C)** Representative images of abnormal parasites quantified in **(B)**. Nuclear (N) and kinetoplast (K) DNA were visualized by DAPI staining (blue). Merge DAPI/DIC images are shown. Statistical analysis: Student’s t-test, *P < 0.05. Scale bars 2 µm.

Given the observed disruption of SUMO conjugation, we next examined its impact on parasite proliferation and cell cycle progression. Growth curve analysis showed that ML-792-treated parasites exhibited an increased doubling time (9.6± 0.5 hours) compared to DMSO-treated controls (8.3± 0.6 hours), indicating impaired proliferation (**Figure 4A**).

To further characterize this phenotype, nuclei and kinetoplast (N/K) configurations were analyzed by DAPI staining (Wheeler et al., 2013), a standard approach to monitor cell cycle stages in *T. brucei*. While all canonical configurations (1N1K, 1N2K, 2N2K) were still observed following treatment, ML-792 exposure altered their relative distribution, with accumulation of cells displaying abnormal N/K configurations (**Figure 4B**).

Defects in nuclear and kinetoplast segregation were frequently detected, often leading to the formation of enlarged or multinucleated cell clusters (**Figure 4C**). These abnormalities are indicative of impaired cell cycle progression and are consistent with defects in mitotic processes.

Together with the reduction in SUMO conjugates, these findings support a model in which ML-792-mediated disruption of the SUMO pathway contributes to defective cell cycle progression and reduced parasite proliferation.

## Discussion

The central role of PTMs, particularly SUMOylation, in the biology of pathogenic trypanosomatids has been well established, positioning this pathway as an attractive target for therapeutic intervention. In this study, we explored the effect of ML-792, a potent and selective inhibitor of the SUMO-activating enzyme, as a strategy to disrupt *Trypanosoma brucei* homeostasis.

The rationale for targeting this pathway is supported by evidence across multiple biological systems. In the parasite *Plasmodium falciparum*, selective inhibition of the SUMO protease *Pf*SENP1 has been shown to block parasite replication both *in vitro* and *in vivo* in infected erythrocytes (Ponder et al., 2011), highlighting the sensitivity of protozoan parasites to disruption of SUMO-dependent processes. In parallel, studies in cancer models have demonstrated that SUMO pathway inhibition preferentially affects highly proliferative cells by impairing mitosis and chromosome segregation (He et al., 2017; Langston et al., 2021). Given that bloodstream form parasites exhibit rapid proliferation relative to most host cells, these observations suggest that *T. brucei* may be particularly vulnerable to pharmacological perturbation of SUMOylation.

Consistent with this hypothesis, ML-792 reduced parasite viability *in vitro* within a pharmacologically relevant concentration range. At the molecular level, treatment resulted in a marked decrease in global SUMO conjugates, with the persistence of some high molecular weight substrates that mirror the phenotype observed upon RNA interference (RNAi)-mediated depletion of SUMO in *T. brucei* (Obado et al., 2011). This concordance between pharmacological inhibition and SUMO knockdown suggests that the residual high molecular weight species represent a stable pool of SUMOylated proteins with slow turnover kinetics. Consistently, this reduction in SUMO conjugates was accompanied by reduced nuclear SUMO signal intensity, indicating effective disruption of SUMO-dependent protein modification. The uniformity of this response across the parasite population further supports the bioavailability and activity of the compound under the conditions tested.

The *T. brucei* genome encodes a single heterodimeric SUMO-activating enzyme, with the SAE2 subunit displaying significant sequence conservation with its human counterpart. In addition, structural analyses revealed preservation of key features within the catalytic region, including the adenylation pocket where ML-792 is known to form a SUMO–inhibitor adduct that blocks enzyme activity. Together, these observations support a model in which ML-792 inhibits SUMOylation in *T. brucei* through a mechanism analogous to that described in mammalian systems. However, direct biochemical validation of target engagement will be required to confirm this hypothesis, for example using recombinant proteins or reconstituted SUMOylation systems (Iribarren et al., 2015b).

While structural conservation predicts similar binding affinity at the active site, cytotoxicity data in human B cells reveal a more nuanced selectivity profile. ML-792 showed minimal toxicity in naive, non-proliferating B cells, whereas it induced significant cytotoxicity in activated B cells and Epstein-Barr virus (EBV)-infected B cells, both of which exhibit high proliferative rates comparable to bloodstream-form *T. brucei* (Garcia et al., 2021). These findings suggest that the therapeutic window for ML-792 may depend less on structural differences between parasite and host enzymes, and more on differential reliance on SUMOylation pathway activity between rapidly dividing pathogens and quiescent mammalian cells.

This “proliferation-dependent selectivity” is consistent with the essential role of SUMOylation in cell cycle progression and DNA replication, processes that are constitutively active in the parasite but largely dormant in non-dividing host cells. Thus, while structural conservation limits classical target-based selectivity, functional selectivity based on cellular proliferation status may provide a viable path forward for antitrypanosomal therapy.

Interestingly, although ML-792 reduced global nuclear SUMO levels, it did not lead to complete disassembly of the HSF, nor did it alter the localization of the ESB, a transcriptionally active Pol I compartment associated with the active VSG expression site. This observation is consistent with the reported stability of the HSF, which is among the last nuclear structures to disassemble during cell cycle progression (Lopez-Farfan et al., 2014). The persistence of this structure under partial SUMO pathway inhibition suggests that localized SUMOylation or structural buffering mechanisms may sustain its integrity. Consequently, under the conditions tested, major alterations in antigenic variation are unlikely, although this possibility should be explored under conditions of stronger or prolonged inhibition.

At the cellular level, inhibition of SUMOylation was associated with impaired proliferation and defects in cell cycle progression, as evidenced by increased doubling time and the accumulation of cells with abnormal nuclei/kinetoplast configurations. These phenotypes closely resemble those observed upon *Tb*SUMO depletion by RNAi, which leads to mitotic defects and aberrant cellular morphology (Obado et al., 2011). This convergence supports the interpretation that the observed effects are primarily driven by disruption of SUMO-dependent processes. In this context, ML-792 provides a complementary approach to genetic perturbation, enabling rapid, tunable, and reversible modulation of SUMOylation levels, and thus represents a valuable tool to investigate SUMO pathway dynamics in *T. brucei*.

From a translational perspective, ML-792 exhibits *in vitro* activity comparable to that of diminazene aceturate, a drug currently used for the treatment of animal trypanosomiasis (Giordani et al., 2016; Dofuor et al., 2019). These findings support further evaluation of SUMO pathway inhibitors in preclinical models. In this context, derivatives such as TAK-981 (subasumstat), which have been optimized for improved pharmacological properties including solubility, bioavailability, and duration of action, represent particularly promising candidates (Langston et al., 2021; Kumar et al., 2022). Preliminary observations in *Trypanosoma cruzi* indicate that ML-792 reduces the viability of epimastigotes but has limited effects on intracellular amastigotes, potentially reflecting constraints in cellular uptake (data not shown). This highlights the importance of compound optimization and delivery in extending the applicability of SUMO-targeting strategies across different parasite stages and species.

In summary, our findings identify the SUMOylation pathway as a viable target in *T. brucei* and provide proof-of-concept for the use of SUMO E1 inhibitors as potential therapeutic agents. These results establish a framework for future studies aimed at validating target engagement, optimizing compound properties, and assessing efficacy *in vivo*, ultimately contributing to the development of new strategies against African trypanosomiasis and related neglected diseases.

## Data Availability

The raw data supporting the conclusions of this article will be made available by the authors, without undue reservation.

## Supplementary material

Supplementary Figure 1 Anti-*Tb*SUMO specificity validation. BSF *Tb*SUMO RNAi expressing parasites (Lopez-Farfan et al., 2014) were used to test *Tb*SUMO antibody specificity. Parasites with (Dox +) or without (Dox -) RNAi induction for 48 h were collected by centrifugation and SUMO conjugates were analyzed by Western blot using polyclonal anti-*Tb*SUMO antibodies in whole cell extracts.

Supplementary Figure 2 Inhibition of SUMOylation by ML-792. BSF parasites were incubated for 48 h in the presence (+) or absence (−) of 5 µM ML-792. Whole-cell extracts were boiled in Laemmli sample buffer immediately after harvesting. Proteins were separated by SDS-PAGE (1x10^7^ cells/lane), and SUMO conjugates were analyzed by Western blot using anti-*Tb*SUMO antibodies. Anti-PABP-C antibodies were used as loading control.
