# Supplementary figures and images for: ML-792 impairs Trypanosoma brucei growth through SUMO pathway disruption: toward E1 as a candidate antitrypanosomal target
Source: Front Cell Infect Microbiol. 2026 Jun 19;16:1850640. doi: 10.3389/fcimb.2026.1850640 (PMC13328426; doi:10.3389/fcimb.2026.1850640)

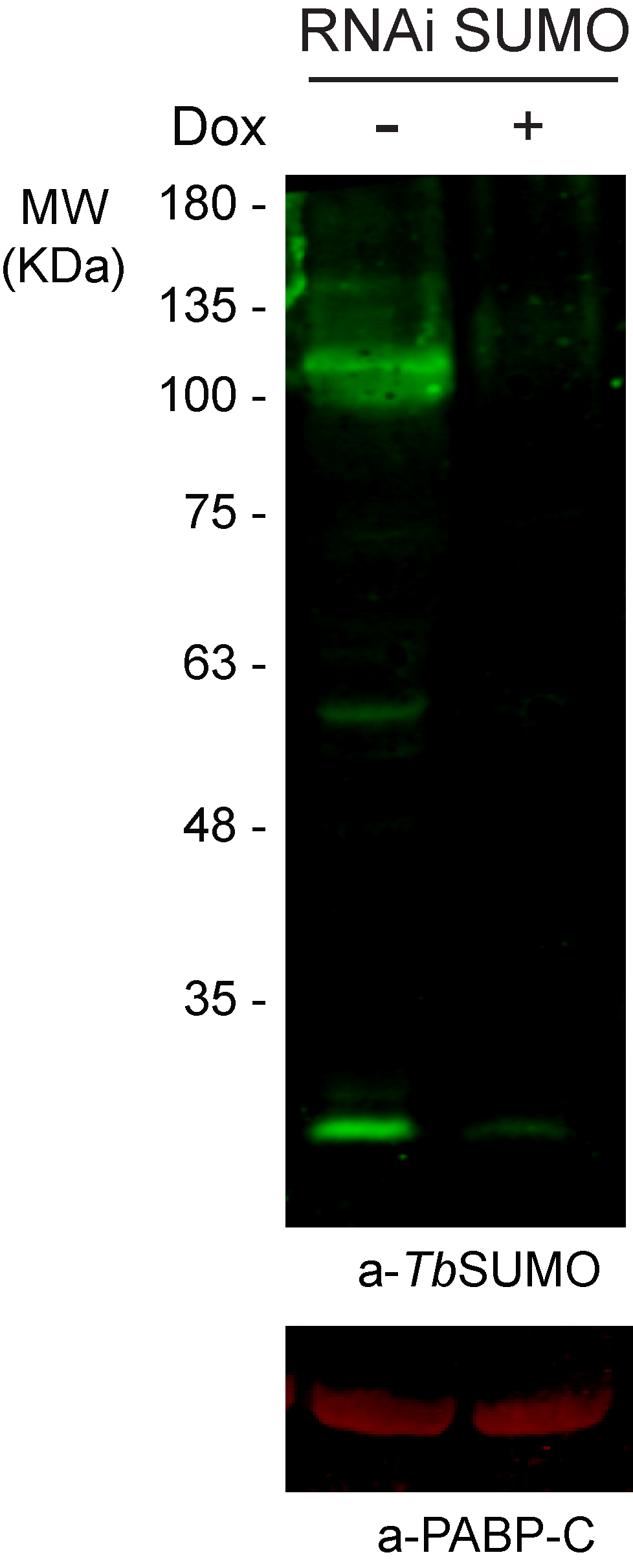

Supplement: Supplementary Figure 1 — Anti-TbSUMO specificity validation. BSF TbSUMO RNAi expressing parasites (Lopez-Farfan et al., 2014) were used to test TbSUMO antibody specificity. Parasites with (Dox +) or without (Dox -) RNAi induction for 48 h were collected by centrifugation and SUMO conjugates were analyzed by Western blot using polyclonal anti-TbSUMO antibodies in whole cell extracts. [file Image1.tif]

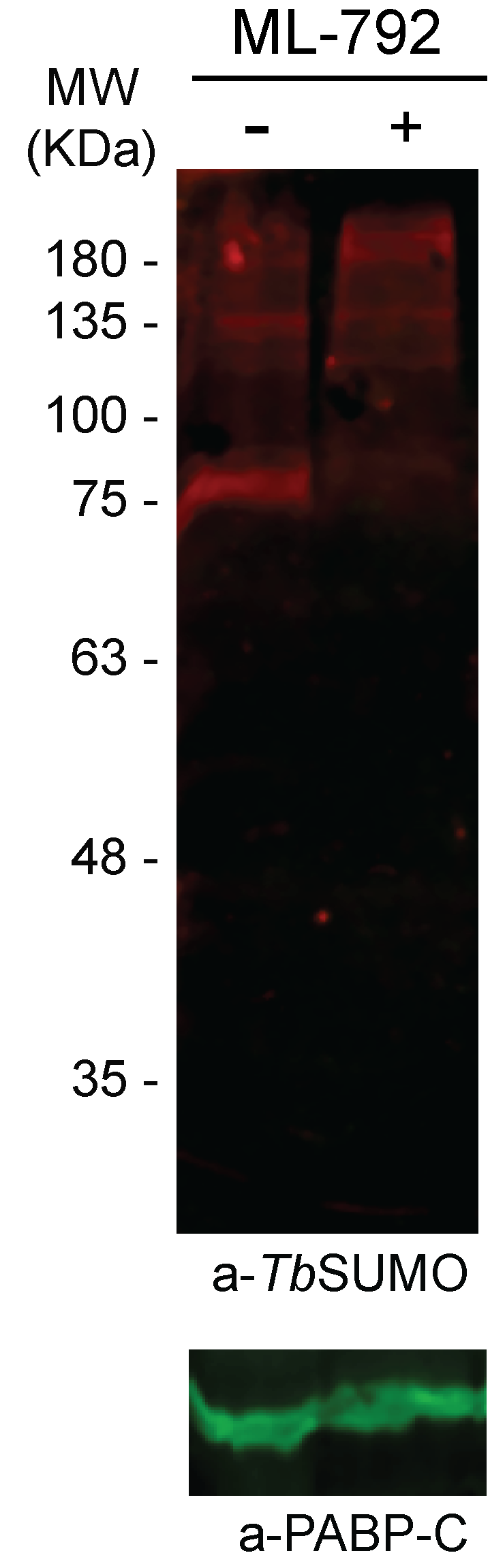

Supplement: Supplementary Figure 2 — Inhibition of SUMOylation by ML-792. BSF parasites were incubated for 48 h in the presence (+) or absence (−) of 5 µM ML-792. Whole-cell extracts were boiled in Laemmli sample buffer immediately after harvesting. Proteins were separated by SDS-PAGE (1x10^7^ cells/lane), and SUMO conjugates were analyzed by Western blot using anti-TbSUMO antibodies. Anti-PABP-C antibodies were used as loading control. [file Image2.tif]
